# Evaluation of trauma care using TRISS method: the role of adjusted misclassification rate and adjusted w-statistic

**DOI:** 10.1186/1749-7922-4-2

**Published:** 2009-01-15

**Authors:** Sadik S Llullaku, Nexhmi Sh Hyseni, Cen I Bytyçi, Sylejman K Rexhepi

**Affiliations:** 1 University Clinical Centre of Kosova, Department of Paediatric Surgery, 10 000 Prishtina, Kosova; 2 University Clinical Centre of Kosova, Department of Orthopaedic and Trauma Surgery, 10 000 Prishtina, Kosova; 3 University Clinical Centre of Kosova, Department of Internal Diseases, 10 000 Prishtina, Kosova

## Abstract

**Background:**

Major trauma is a leading cause of death worldwide. Evaluation of trauma care using Trauma Injury and Injury Severity Score (TRISS) method is focused in trauma outcome (deaths and survivors). For testing TRISS method TRISS misclassification rate is used. Calculating w-statistic, as a difference between observed and TRISS expected survivors, we compare our trauma care results with the TRISS standard.

**Aim:**

The aim of this study is to analyze interaction between misclassification rate and w-statistic and to adjust these parameters to be closer to the truth.

**Materials and methods:**

Analysis of components of TRISS misclassification rate and w-statistic and actual trauma outcome.

**Results:**

The component of false negative (FN) (by TRISS method unexpected deaths) has two parts: preventable (Pd) and non-preventable (nonPd) trauma deaths. Pd represents inappropriate trauma care of an institution; otherwise nonpreventable trauma deaths represents errors in TRISS method. Removing patients with preventable trauma deaths we get an Adjusted misclassification rate: (FP + FN - Pd)/N or (b+c-Pd)/N. Substracting nonPd from FN value in w-statistic formula we get an Adjusted w-statistic: [FP-(FN - nonPd)]/N, respectively (FP-Pd)/N, or (b-Pd)/N).

**Conclusion:**

Because adjusted formulas clean method from inappropriate trauma care, and clean trauma care from the methods error, TRISS adjusted misclassification rate and adjusted w-statistic gives more realistic results and may be used in researches of trauma outcome.

## Background

Major trauma is defined as a severe trauma injury when the patient dies in ED or needs major surgical operation on the head, chest, abdomen or inguinal areas or needs immediate ICU admission [1]. If ISS > 15 major trauma is considered. The incidence of major trauma is around 340 – 522 in one million inhabitants per year, and mortality is still high [2,3]. Trauma patients occupy more hospital beds then all patients from heart diseases, and four times more than patients with cancer [4]. Most often are locomotors injuries, but the main cause of death is head trauma [5-7]. Trauma is still the leading cause of deaths of children in industrialized countries [8]. The rate of preventable trauma deaths in the literature is 30% in nontrauma hospitals, and 1 – 5% in trauma centers. In the past two decades of trauma literature the scoring systems issues are very actual; the three most citied articles in the Journal of Trauma are from the field of trauma scoring [9].

Trauma injury produces body damages. The most famous anatomical trauma scoring systems are AIS (Abbreviated Injury Scale) and OIS (Organ Injury Scaling). In AIS injuries are scaled from 1 (minor) to 6 (unsurvivable) [10-12]. Injury Severity Score (ISS), published by Baker in 1974 [13,14]. is anatomic scoring system, which takes on consideration the three major injuries in different body regions, but using only the highest AIS value on the specific region.

It identifies all anatomical injuries (from clinical examination, imagery examinations, surgical procedures or autopsy) on six body regions: 1. Head and neck, 2. Face, 3. Chest, 4. Abdomen, 5. Extremities (including pelvic bones), 6. External. Calculating formula: ISS = (AIS_1_)^2 ^+ (AIS_2_)^2^+ (AIS_3_)^2^.

The ISS value goes from 0 to 75. If, in any organ we have AIS injury = 6 (unsurvivable) then we have a value of ISS = 75. The higher are the ISS values the more serious the trauma is. The physiological derangements created by trauma are evaluated with RTS (Revised trauma score) [15] when patients is arriving in ED. ISS and RTS are the main components of TRISS method Trauma Score – Injury Severity Score (TRISS) is widely used method to predict probability of survival (P(s) [16] based on formula: P_(s) _= 1/(1+ e^-b^) e = 2.718282 (base of natural logarithm), b = b_0 _+ b_1 _(RTS) + b_2 _(ISS) + b_3 _(Age index). For patients under 55 years old, the age index is = 0, but for patient > 55 years old the age index is = 1. The coefficients b0, b_1_, b2, b3 are produced from multiple regression analyses from database. For patients less than 15 years of age only the values of non penetration type of injuries are taken. To calculate P(s) TRISS calculator is used ; .

TRISS method is assessed analyzing: sensitivity, specificity, positive predictive value (PPV), Negative predictive value (NVP), false positive, false negative, and misclassification rate. The misclassification rate represents the sum of false positive and false negative values as percentage and is considered to be the best index of general value of TRISS [17] When we evaluate the in-hospital trauma care using TRISS method, usually is calculated W – statistic which represents the number of survival patients more or less than the norm of TRISS method, using the formula: W = 100 * [(observed survivals) - (predicted to survive)]/total number of patients.

## Aim

The aim of this study is to analyze interaction between TRISS misclassification rate and w-statistic and to adjust these parameters to be closer to the truth when we evaluate predicted and observed trauma outcome.

## Methods

When trauma outcome and trauma care is evaluated with TRISS method and wstatistic is compared with the standard a question is raised: Is the mirror's fault for the face reflection? Then the needs accrue to face the correctness of the method (the misclassification rate) with the correctness of trauma care (w-statistic rate). This is achieved when from the calculation of misclassification rate preventable deaths are removed (observed deaths, but by TRISS method predicted to survive and by audit considered as preventable trauma deaths), and on other hand no preventable deaths are eliminated form w-statistic (observed deaths, but by TRISS method predicted to survive and by audit considered as non preventable trauma deaths).

While misclassification rate of TRISS method is valued as a sum of false positive values and false negative values divided with total number of analyzed patients, (FP+FN)/N, or (b+c)/N, or as the sum of unexpected survivors and unexpected deaths and divided with general number of patients (Us +Ud)/N, the adjusted misclassification rate it considers also the preventable deaths (Pd = c - nonPd), therefore when this component is incorporated in the misclassification rate formula, we will get the adjustment which results in lower value of the misclassification rate: (b+c - Pd)/N), respectively (FP+FN - Pd)/N, respectively (Us +Ud - Pd)/N. In other words TRISS method is "clean" from preventable trauma deaths which depend on the hospital trauma care quality rather the validity of the TRISS method. That's the way the adjusted rate of misclassification can be lower than the misclassification rate. The adjusted rate of misclassification represents the real correctness of method. This can be illustrated with one example case: if one patient has spleen rupture with AIS 3 and he arrives in hospital in normal vital parameters the TRISS method will predict survival for such patient; and if this patients dies in hospital due to haemorrhagic shock, this case study is classified as preventable death.

W-statistic stand for the difference between the number of patients that has actually survive and those that TRISS method has predicted to survive in percentage: (Os - Es)/N; respectively: {(b + d) - (c + d)}/N or (b - c)/N, or (FPFN)/N.

When the **c **(FN or Ud) values is analyzed we notice that there are two components: preventable deaths (Pd) and non preventable deaths (nonPd – TRISS expected survivors, but clinically no preventable deaths).

The **b **(FP or Us) component which represents the number of false positive mistakes respectively the unexpected survivors; it's desirable to be higher than zero and represents more survivors than the TRISS methodology standard. While preventable deaths are a result of lack of trauma care, the non preventable deaths represents the lack of the method and should be incorporated in a (TP) value, respectively in the group of actually observed and expected deaths.

So from the w-statistic rate should be eliminated nonPd component of **c **(FN) value therefore we gained adjusted w-statistic: from w-stat = (b-c)/N from c value we subtract nonPd: {b - (c - nonPd)}/N or (b - c + nonPd)/N.

Because c - nonPd = Pd, it can be also written: (b - Pd)/N, or (FP-Pd)/N. (Table 1).

**Table 1:** Adjusted TRISS misclassification rate and adjusted w-statistic

TRISS Method	deaths Od	survivors Os	Expected:
Positive (P(s) < 50%)	TP	FP	Ed = TP+FP
	**a**	**b**	Ed = a+b
	EOd	Us	Ed = Us+ EOd

Negative (P(s) > 50%)	FN	TN	Es = FN+TN
	**c = c1+c2**	**d**	Es = c+d
	**c1 = Pd;**	EOs	Es = Ud+EOs
	**c2 = nonPd**		
	Ud		

Observed:	Od = TP+FN	Os = FP+TN	N = Ed+Es
	Od = a+c	Os = b+d	N = a+b+c+d
	Od = EOd+Ud	Os = Us+EOs	N = Od+Os

Misclassification rate = (**FP + FN)/N **= (b + c)/N = (Us + Ud)/N			

Adjusted misclassification rate		= **(FP+FN-Pd)/N**	
		= (b +c -Pd)/N	
		= Us + Ud - Pd/N	

W statistic		= (FP+TN) - (FN+TN)/N = **(FP - FN)/N**	
		= (b+d) - (c+d)/N = (b-c)/N	
		= (Os - Es)/N	
		= (Us+EOs) - (Ud + EOs)/N = (Us - Ud)/N	

Adjusted W statistic		= [FP-(FN-nonPd)/N = **(FP- Pd)/N**	
		= [b - (c - nonPd)]/N = (b - Pd)/N	
		(c = c1+ c2; **Pd = c - nonPd**)	
		= (Us - Pd)/N	
		= [Os- (Es- nonPd)]/N	

Elements of TRISS misclassification rate and w-statistic, formulas and abbreviation used to describe observed and expected outcome Abbreviations: TP (a) = True positive = EOd = Expected observed deaths, FP (b) = False positive = Us= Unexpected survivors, FN (c) = False negative = Ud = Unexpected deaths, (c1) = Pd = preventable deaths, (c2) = nonPd = Nonpreventable deaths, TN (d) = True negative = EOs = Expected observed survivors, Ed= Expected deaths, Es = Expected survivors, Od = Observed deaths, Os = Observed survivors.

## Results

The adjusted TRISS misclassification rate: (b+c - Pd)/N), respectively (FP+FN - Pd)/N, respectively (Us + Ud - Pd)/N. If b = FP = 0 (no unexpected survivors) than: (c-Pd)/N) = (FN-Pd)/N, respectively:nonPd/N.

Adjusted w-statistic: (b - Pd)/N, or (FP-Pd)/N, respectively [(Os-Es) +nonPd]/N.

If nonPd > 0 then also the final result of adjusted w-statistic appears improved (less negative, zero or positive) than w- statistic. This adjustment creates a more correct value which is closer to the true quality level of trauma care in those institutions where the evaluation with this method is taking place.

When b = FP = O (no unexpected survivors) than the adjusted w-statistic represents the negative value of preventable deaths: (-Pd/N) (Table 1).

Examples:

1. In ideal case the misclassification rate and the w-statistic should have zero value (O): a = 30, b = 0, c = 0, d = 70, Misclassification rate(b+c)/N = (0+0)/100 = 0%; w-statistic = (b-c)/N = = (0-0)/100 = 0%.

Trauma care is excellent compared to standard, and method perfectly predicts who will survive and who will die.

2. Commonly in developing countries we may find such situation:

a = 30, b = 0, c = 15, d = 55

Misclassification rate = (b+ c)/N = (0+15)/100 = 15% (misclassification rate is so high: is method weak?) and w-stat = (b-c)/N = (0–15)/100 = -15% (deeply negative: is inappropriate trauma care ?)

a) If all unexpected deaths are preventable deaths (FN = c = c1 = Pd) than:

Adjusted misclassification rate = (b+c-Pd)/N = (0 +15-15)/100 = 0%!

Adjusted w-stat = b - Pd = (0 –15)/100 = - 15% remains the same.

The method is perfectly predicting outcome, but the trauma care is insufficient.

The mirror is not to blame for the face reflection!

b) If all unexpected deaths are no preventable trauma deaths (FN = c = c2= nonPd; Pd = 0) than:

Adjusted misclassification rate: (b+c-Pd)/N = 0+15-0)/100 = 15% and Adjusted w- stat = b- Pd = (0 - 0)/100= 0%!

So, the trauma care is as good as the standard but the method is wrong: its mirror's fault for the face reflection!

3. Analyzing trauma outcome in 2002 in our hospital we found that from 163 major traumas actually 90 have survived, 73 have died, while by TRISS method 124 have been expected to survive, and 39 to die. All expected to die already have died (Table 2). So: a = 39, b = 0, c = 34, d = 90.

**Table 2:** Adjusted TRISS misclassification rate and adjusted w-statistic in 163 major trauma

TRISS Method	deaths	survivors
	
	Od	Os
Positive (P(s) < 50%)	TP	FP
	**a = 39**	**b = 0**
	EOd	Us

Negative (P(s) > 50%)	FN	TN
	**c = 34**	**d = 90**
	**c1 = Pd = 30**	EOs
	**c2 = nonPd = 4**	
	Ud = Pd+nonPd	

Differences between misclassification rate and w-statistic and adjusted misclassification rate and adjusted W statistic where Pd are identified. Adjusted misclassification rate drops from previous 20.8% on reliable 2.4%.

Misclassification rate = (b + c)/N = (0+34)/163 = 20.8%

Adjusted misclassification rate = (b+c-Pd)/N = (0+34-30)/163 = 4/163 = 2.4%

W statistic = (b-c)/N = (0–34)/163 = -20.8%

Adjusted w- statistic = (b - Pd)/N = (0–30)/163 = -18.4%

Misclassification rate = (b+ c)/N = (0+34)/163 = 20.8% and W-stat = (b-c)/N = (0–34)/N = -20.8%.

Auditing unexpected deaths (FN = c value) we considered that c1 = Pd = 30 and c2 = nonPd = 4, so:

Adjusted misclassification rate = (b+c-Pd)/N = (0+34-30)/163 = 2.4%!

Adjusted w-stat = (b - Pd)/N = (0–30)/163 = -18.4%.

The method offers almost realistic trauma outcome prediction (misclassification rate significantly drops from 20.8% to 2. 4%), but there is a trauma care lack (w -statistic despite adjustment still is deeply negative: -18.4) and the method cannot blamed. The mirror is not to blame for the face reflection!

## Discussion

All over the world the traumatic injuries are still remaining as one of the major problems in health and social issues in general and the leading cause of death worldwide. Trauma as an unexpected attacker with serious and fast anatomic and physiological consequences for the individual, which often can be fatal in short period of time, especially in prehospital phase, up till now the mortality rate in hospital from trauma injuries still remain high with 7–45% [18]

Unexpected deaths (Ud) are the object of analysis of trauma care quality. On the other hand the unexpected survivors (Us) are welcomed and reflect trauma care above the methods standard. Unexpected deaths (Ud) often correspond to as insufficient trauma care.

There are few of trauma centers that with their practice have achieved higher results then the actual standard – meaning that they have unpredictable survivors based on TRISS method. There are more publications on TRISS presenting considerable percentage of unpredictable deaths.

Norris R and al. from Level I trauma centre have published that 2.5% amongst trauma patients treated there have been TRISS unexpected survivors [19] West and Trunkey (1979) have documented that 2/3 deaths from non -brain injuries and 1/3 deaths from brain injuries has been preventable in regions with no trauma centers [20] TRISS method is widely used in evaluating the trauma outcome, it defines the probability of survival and it is used as a standard for evaluating the quality of trauma care in hospitals. TRISS methodology is also applicable in evaluating children traumas [18]. Based in this method the w-statistic is calculated as percentage of the difference of actual survivors and predicted survivors. The discrepancy between predicted trauma outcome and the observed outcome of studied population depends on correctness of the method, and on the real quality of the trauma care. The unexpected survivors(Us) of TRISS method present the methods error, but in practice they are more than welcomed because they presents better result than the standard, wile unexpected deaths(Ud) represents undesirable outcome, loss of patients which the method has predicted to survive. In this group of patients classified by TRISS method as false negative values two sub-groups are defined: preventable trauma deaths (Pd) and non- preventable trauma deaths (nonPd). Knowing this subgroups we are able to calculate adjusted TRISS misclassification rate and adjusted w-statistic. Preventable trauma deaths are clinical reality, but the ways for identification of preventable trauma deaths still are not standardized and need to improve [21]. Besides some critics and objective limitation, TRISS method still remains the most used method in trauma outcome studies. [5]

## Conclusion

In many studies trauma outcome inevitable imposes as a key element for evaluation and comparison of the results between different institutions or their maturity. TRISS method has proven to have an important role in trauma care research. While the group of unexpected survivors (Us) is do to methods error, the group of patients with unexpected deaths (Ud) has two sub-groups: Pd and nonPd. Pd represents inappropriate trauma care of an institution; otherwise nonpreventable trauma deaths represents errors in TRISS method.

So, evidencing those two subgroups it is possible to adjust the values of w-statistic and the values of the misclassification rate. Because the adjusted formulas cleans the method from inappropriate trauma care and clean trauma care from the methods error, TRISS adjusted misclassification rate ((FP+FN - Pd)/N, and adjusted w-statistic ((FP-Pd)/N) give more realistic results and are useful in the research of trauma care evaluation.

## Competing interests

The authors declare that they have no competing interests.

## Authors' contributions

SSL carried out design of the study, drafted the manuscript and performed statistical analysis. NSH participated in design of the study and in drafting the manuscript. CIB participate in design of the study and in drafting the manuscript. SKR participated in drafting manuscript and statistical analysis. All authors read and approved the final manuscript.
